# Evaluation of two single-factor models of metabolic syndrome: a confirmatory factor analysis for an adult population in Beijing

**DOI:** 10.1186/1476-511X-12-61

**Published:** 2013-05-02

**Authors:** Da Huo, Wei Wang, Xia Li, Qi Gao, Lijuan Wu, Yanxia Luo, Youxin Wang, Puhong Zhang, Xiuhua Guo

**Affiliations:** 1 School of Public Health, Capital Medical University, No.10 Xitoutiao, You'anmen Wai, Beijing, Fengtai District, 100069, China; 2 Beijing Municipal Key Laboratory of Clinical Epidemiology, Beijing, 100069, China; 3 Department of Integrated Early Childhood Development , Capital Institute of Pediatrics, No.2 Yabao Rd, Beijing, Chaoyang District, 100020, China; 4 The George Institute for Global Health at Peking University Health Science Center, Level 18, Tower B, Horizon Tower, No.6 Zhichun Rd, Beijing, Haidian District, 100088, China

**Keywords:** Metabolic syndrome, Confirmatory factor analysis, Single-factor model, Chinese

## Abstract

**Background:**

Prevalence of metabolic syndrome is high and increasing in China. The causation of this disorder is, yet, to be fully understood. Several studies with confirmatory factor analysis have been performed to investigate the core of the disease in some races other than Chinese, and amongst the other studies, they have yielded a sound model fit. This study was to evaluate and compare two single-factor models of the underlying factor structure of metabolic syndrome in a Chinese population using confirmatory factor analysis.

**Results:**

Findings showed that in a Chinese sample of 7,472 individuals, Model 1 (with waist circumference, triglycerides/HDL-C ratio, fasting plasma glucose and mean artery pressure) yielded good level of fitness (SRMR < 0.08, CFI > 0.96 and RMSEA < 0.10) in men and women of all age groups; and Model 2 (with waist circumference, triglycerides, fasting plasma glucose and systolic blood pressure) fitted well in men aged 18–34 and over 60 and in all women, except in men of 35–59 (RMSEA = 0.142). In comparison, Model 2 were shown to be better fit (with relative larger GFI and smaller AIC, BIC, CAIC, and EVIC) in women of all age groups and in men of 18–34 and over 60 years old; Model 1 had a better fit in men between 35 and 59.

**Conclusions:**

This study suggests that the single-factor model of metabolic syndrome with waist circumference, triglycerides, fasting plasma glucose and systolic blood pressure are plausible in women of all age groups and young and senior men in Beijing. The model with waist circumference, triglycerides/HDL-C ratio, fasting plasma glucose and mean artery pressure fits middle-aged men.

## Background

The metabolic syndrome (MetS) is conceptualized as a constellation of multiple, closely-related metabolic disorders. It is a major global public health problem in both developed and developing countries [1]. The commonly encompassed features of MetS are insulin resistance, hypertension, abdominal obesity, and dyslipidemia [1-4]. Those seemingly unrelated biological processes have been proved to occur at a frequency higher than by mere chance. In 1988, Reaven proposed an underlying pathophysiological causation and named it as *Syndrome X*[5]. And the syndrome appears to increase the risk of developing cardiovascular disease and type 2 diabetes mellitus [6-9]. Although, it has been known for at least eighty years [10], the definition for MetS has been developing with time – World Health Organization (WHO) in 1999 [11], International Diabetes Federation (IDF) in 2004 [2], US National Cholesterol Education Program (NCEP) Adult Treatment Panel III (ATP III) in 2001 [12] and a modified edition in 2005 [13]. In China, Chinese Diabetes Society (CDS) [14] and Joint Committee for Developing Chinese Guidelines on Prevention and Treatment of Dyslipidemia in Adults (JCDCG) [15] also released their diagnosing criteria specifically for the Chinese population in 2004 and 2007, respectively. In 2009, a joint interim statement by six major institutions was released [16]. In the statement, raised waist circumference (WC), elevated triglycerides (TG), reduced high-density lipoprotein cholesterol (HDL-C), elevated systolic and/or diastolic blood pressure (SBP, DBP), and elevated fasting plasma glucose (FPG) were included in the diagnosis criteria. WC, the indicator for central obesity, is defined as population- or country-specific.

Though the definition has been agreed upon, the mechanism of MetS is still controversial [17], such as some declaring that insulin resistance might be the major cause [5,18]. There is debate about the essence of the MetS pertaining to which components are included and what pathologic process is central to its occurrence. The commonly included components are hypertension, obesity, elevated blood glucose, and dyslipidemia. These factors tend to cluster as a risk factor for the morbidity of cardiovascular disease, type 2 diabetes mellitus, and overall mortality [1,13].

In recent years, factor analysis has been applied to shed some light on finding the “common soil” for the syndrome [19]. Exploratory factor analysis (EFA), a multi-factorial statistical procedure, is used to extract a relatively small set of latent variables from the extensively observed ones. Observed variables are directly measurable, while the latent are the underlying factors. Studies with EFA indicate differences in the number of factors extracted and the variable loadings on each factor. The inconsistence may be due to the nature of EFA and the methods applied in the extraction of variables. The variables shared in common are assumed to be the underlying latent variables [20].

Confirmatory factor analysis (CFA) is another way to evaluate the factor structures of MetS based on the theoretical foundations set by EFA [21]. It is used to analyze one or more latent causative factors underlying a concept, i.e. MetS in our study, by comparing the distribution and the established factor structure based on the known concept [22].

With a *priori* selected factor models from previous research, CFA can be used to compare competing models of MetS using the same dataset to determine which of the two or more hypothesized models fits best [23].

The aim of this study is to evaluate and compare two competing models of metabolic syndrome using CFA in a Chinese population. There are two single-factor models for candidate: Model 1 is by Pladevall *et al.* and Martinez-Vizcaino [24,25], with WC, TG/HDL-C ratio, and mean arterial pressure (MAP) as factors, but HOMA-IR (homeostasis model of assessment for insulin resistance) or fasting insulin in the original models is substituted by fasting plasma glucose referred to the latest diagnosis criteria for MetS [16]; Model 2 is presented by Li and Ford [26] with WC, TG, and SBP, while fasting insulin is substituted by FPG.

## Results

### Population profile

There were 16,711 individuals (87.0% of the total 19,216 subjects we selected) who finished the questionnaire (6,658 men, 45.83 ± 14.47 years; 10,053 women, 40.77 ± 12.13 years; gender ratio: female/male = 1.51). There were 688 subjects with no anthropometric, physiologic, or blood biochemical characteristic measurements were excluded. Afterwards, 8,551 people with anti-hypertensive, anti-dyslipidemic, or anti-hyperglycemic treatment were excluded. Therefore, 7,472 subjects were finally used for the analysis (2,666 men, 40.83 ± 14.47 years; 4,806 women, 40.77 ± 12.13 years), and gender ratio female/male was 1.80.

About 51.7% of male participants smoked every day versus only 2.9% in female. And 41.1% of men drank alcohol at least once a week, versus 3.3% of women. According to the last definition for MetS in 2009 [16], elevated WC was observed in 28.7% in men and 34.9% in women, elevated TG was seen in 23.4% of men and 10.5% of women; low HDL-C was detected in 15.9% of men and 35.7% of women; elevated blood pressure was observed in 46.3% of men and 29.3% of women; elevated FPG was seen in 21.5% of men and 15.9% of women. The prevalence of MetS, the cluster of three or more metabolic risk factors, was 21.5% in men and 16.9% in women. The basic characteristics of the subjects were shown in Table 1.

**Table 1:** Means and standard deviations of physiological and anthrometric characteristics (n = 7,472)

**Measures**	**Male (n = 2,666)**		**Female (n = 4,806)**	
	**Mean**	**SD**	**Mean**	**SD**
Height (cm)	169.8	6.4	159.1^#^	5.6
Weight (kg)	70.0	11.0	59.8^#^	9.2
Waist Curriculum (cm)	84.5	9.7	76.8^#^	9.3
Hip Curriculum (cm)	97.0	6.9	95.5^#^	7.3
Body Mass Index (kg/m^2^)	24.2	3.3	23.6^#^	3.5
Waist/hip Curriculum Ratio	0.87	0.06	0.80^#^	0.06
Systolic Blood Pressure^*^ (mmHg)	128.8	15.6	120.8^#^	17.1
Diastolic Blood Pressure (mmHg)	80.3	10.3	76.9^#^	10.1
Mean Artery Pressure^*^ (mmHg)	96.5	11.2	91.5^#^	11.8
Fasting Plasma Glucose^*^ (mmol/L)	5.29	1.05	5.14^#^	0.83
Total Cholesterols (mmol/L)	4.56	0.94	4.53	0.86
Triglyceride^*^ (mmol/L)	1.36	1.14	1.02^#^	0.76
HDL-C (mmol/L)	1.27	0.31	1.42^#^	0.30
LDL-C (mmol/L)	2.97	0.89	2.85^#^	0.82
TG/HDL-C Ratio^*^	1.22	1.47	0.80^#^	0.85
Creatinine (µmol/L)	83.86	14.23	66.4^#^	11.1

^*^ These values were log_e_ transformed in confirmatory factor analysis;

^#^ These values are significant at *P* < 0.01 compared with male counterparts;

SD, standard deviation.

### Confirmatory factor analysis (CFA)

**Figure 1:**
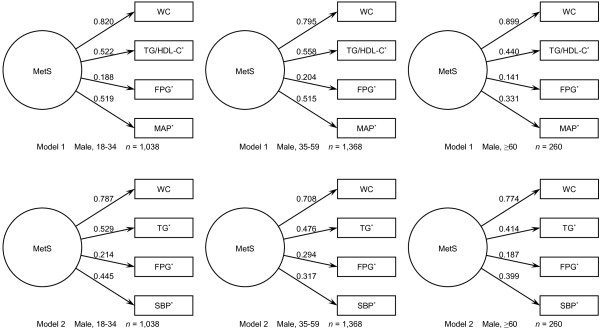
**Two single-factor models for MetS for men in different age groups.** Abbreviations: MetS, metabolic syndrome; WC, waist circumference; TG/HDL-C, the ratio between triglyceride and high-density lipoprotein cholesterol; FPG, fasting plasma glucose; MAP, mean arterial pressure; TG, triglyceride; SBP, systolic blood pressure. Models are grouped in different age ranges in both sexes. Values with asterisk (*) were log_e_ transformed in CFA.

**Figure 2:**
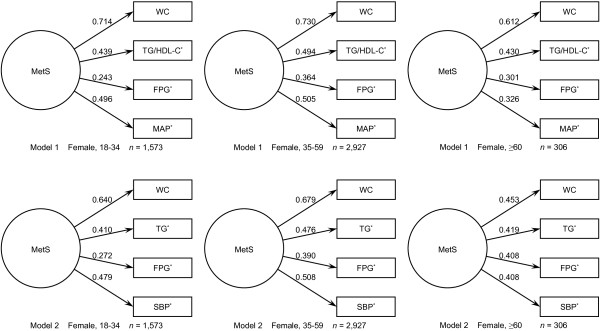
**Two single-factor models for MetS for women in different age groups.** Abbreviations: MetS, metabolic syndrome; WC, waist circumference; TG/HDL-C, the ratio between triglyceride and high-density lipoprotein cholesterol; FPG, fasting plasma glucose; MAP, mean arterial pressure; TG, triglyceride; SBP, systolic blood pressure. Models are grouped in different age ranges in both sexes. Values with asterisk (*) were log_e_ transformed in CFA.

CFA was performed with AMOS v7.0, and the loadings for the factors in each model are shown in Figure 1 and Figure 2. Factor loadings were required to be greater than 0.30 and statistically significant (*P* < 0.05). In both models, all factor loadings were statistically significant. In Model 1, WC was the highest loading among the four factors, while FPG was the least. The loadings of TG/HDL-C ratio and MAP were very close. TG/HDL-C ratio was a bit higher than MAP in men and senior women, while this was opposite in young and middle-aged women. In Model 2, WC was also the highest loading factor. TG was at the second place in men and in young and senior women; while SBP was the second highest loading factor in middle-aged women. FPG had the lowest score in both men and women.

Table 2 showed the fitness of models in each sex-age group. A *χ*^2^ test was used to evaluate if the hypothesized models fit the sample population. In either Model 1 or Model 2, the *χ*^2^ test had a *P* > 0.05 in men and women aged above 60. This indicated that the two single-models did not explain all of the relations among the measured variables in other age groups. However, as the *χ*^2^ test is prone to show a significant lack of model fit in studies with large sample size (over 1000 subjects), it was foreseeable that large *χ*^2^ value might be produced, and its results cannot be merely assessed in isolation. As a result, other indices were introduced. A model was considered to have a good fit when the CFI is more than 0.960 [27] and the SRMR is less than 0.080 [27]. The SRMR (standardized root mean square residual) was less than 0.080 in all age groups in Models 1 and 2. In Model 1, CFI (comparative fit index) was greater than 0.960 in male, and in female aged less than 60; in Model 2, CFI was plausible in every group except men aged 35–59. A model has a poor fit if RMSEA (root mean square error of approximation) is greater than 0.100; a mediocre fit is 0.080-0.100; and 0.050-0.080 means a reasonable fit; while a model has a good fit when the RMSEA is less than 0.050 [27]. According to this criteria, only Model 2 had a poor fit in middle-aged men.

**Table 2:** Summary of statistics and model fit indices

**Model 1**							
	***χ***^**2**^	**df**	***P *value**	**SRMR**	**CFI**	**RMSEA**	**90% CI RMSEA**
Male							
18-34	4.976	2	0.083	0.0212	0.991	0.045	<0.001, 0.096
35-59	8.255	2	0.016	0.0229	0.987	0.055	0.020, 0.096
≥60	3.910	2	0.142	0.0344	0.973	0.061	<0.001, 0.151
Female							
18-34	12.617	2	0.002	0.0226	0.977	0.058	0.030, 0.091
35-59	38.287	2	<0.001	0.0263	0.969	0.079	0.058, 0.101
≥60	5.066	2	0.079	0.0334	0.938	0.071	<0.001, 0.151
**Model 2**							
	***χ***^**2**^	**df**	***P *value**	**SRMR**	**CFI**	**RMSEA**	**90% CI RMSEA**
Male							
18-34	7.047	2	0.030	0.0206	0.987	0.049	0.013, 0.091
35-59	57.212	2	<0.001	0.0516	0.849	0.142	0.112, 0.175
≥60	3.892	2	0.143	0.0318	0.968	0.060	<0.001, 0.150
Female							
18-34	8.579	2	0.014	0.0187	0.982	0.046	0.018, 0.079
35-59	28.680	2	<0.001	0.0229	0.975	0.068	0.047, 0.090
≥60	3.107	2	0.212	0.0260	0.976	0.043	<0.001, 0.129

In Table 3, fit indices were compared between the two competing models. GFI (goodness-of-fit index) showed how well a theoretical matrix could explain the matrix from a data sample. GFI ranges from 0 to 1, and a higher score means a better fit [27]. Generally, GFI of more than 0.90 indicates a good model fit. Both the models in every age group had a sound GFI value. AIC (Akaike information criteria) takes into account the number of parameters of models under evaluation. It is an index to compare models with different latent variables. Models with a smaller AIC indicates a better fit [27,28]. Model 2 had a relative smaller value of AIC in every age group than Model 1, except in middle-aged men. BIC (Bayes information criterion) is similar to AIC in that both are derivatives of an information-theory based test. CAIC (consistent version of AIC) takes into the consideration of sample size effect. ECVI (expected cross-validation index) concerns the overall error. The indices above were used to compare fitness in different models [27]. Model 2 had a relative higher GFI and smaller AIC, BIC, CAIC, and ECVI than Model 1 in women over 35 and men in 18–34 and above 60; while in men of 35–59, Model 1 had a better fit in all set of indices. For young women, Model 2 had a larger GFI, smaller AIC and ECVI, whereas Model 1 had a smaller BIC and CAIC.

**Table 3:** Summary of models fit indices for two competing models

**Model 1**						
	**GFI**	**AIC**	**BIC**	**CAIC**	**ECVI**	**90% CI ECVI**
Male						
18-34	0.996	24.255	63.816	71.816	0.023	0.018, 0.036
35-59	0.986	58.367	100.135	108.135	0.043	0.030, 0.061
≥60	0.993	19.910	48.396	56.396	0.077	0.069, 0.115
Female						
18-34	0.996	28.617	63.095	71.503	0.018	0.013, 0.028
35-59	0.994	54.287	102.141	110.141	0.019	0.013, 0.027
≥60	0.992	21.066	50.855	58.855	0.069	0.059, 0.104
**Model 2**						
	**GFI**	**AIC**	**BIC**	**CAIC**	**ECVI**	**90% CI ECVI**
Male						
18-34	0.997	23.047	62.607	70.607	0.022	0.018, 0.034
30-59	0.981	73.211	114.981	122.981	0.054	0.038, 0.074
≥60	0.993	19.892	48.377	56.377	0.077	0.069, 0.115
Female						
18-34	0.997	24.579	67.465	75.465	0.016	0.012, 0.024
35-59	0.995	44.680	92.534	100.534	0.015	0.011, 0.023
≥60	0.995	19.107	48.895	56.895	0.063	0.059, 0.092

## Discussion

Our results show that although in the same ethnic group, people from different sex-age groups must not be confirmed as one defined model. A number of published CFA studies have tested various hypothetical models, including 1-factor, 2-factor, 4-factor and second-order latent factor models [19-22,24], but there continues to be debate regarding which model best represents the factor structure for underlying cardiovascular risk factor clustering. But as a syndrome, there might be a core for the obvious disorders, and the single-factor model would be preferable.

In our study, WC is the leading factor for MetS. MetS is based on insulin resistance, however, the HOMA-IR is not available for this study, and WC is not the best but the most used manifest for that. It is consistent with former studies in other populations [24,29]. The models applied in different age groups have a great deal to do with the ultimate fit of the model. Regardless of how well specified the model is or its goodness of fit, factor analysis does not and cannot “prove” trait clustering or its physiological mechanism. Even confirmatory analyses are a function of a hypothetical model. In theory, there are an infinite number of alternative models that could fit data equally well or better and thereby produce the same covariance matrix. This is known as the equivalent models problem in structural equation modeling. Equivalent models are of particular concern for metabolic syndrome research and theory because such equivalent models may produce conflicting interpretations. In this study, Models 1 and 2 are examples of such conflicting models. In our study, Model 2 fitted the data in male of 18–34 and over 60 and in female of all age-groups, whereas Model 1 fitted male subjects of 35–59.

A prior CFA study had tested models similar to 1 and 2, and found it to be valid and invariant across race and sex groups [24]. The model stability of MetS has been examined across sex [20,26], ethnicity [20,26], and age in a male population [30]. It seems that the theoretical model of MetS is consistent with few exceptions. In our study, the models fit differently in specific sex-age group. The concept of a single underlying factor that influences the expression of all observed traits is plausible. However, further longitudinal investigations are needed to explore the invariance of measurement, and to test the model structure stability with time.

This study indicated that the factor structure underlying the clustering of MetS in adults is varied in a population as Beijing’s. The lack of fit and instability of the two models presented indicated that there might be variable components in the structure of MetS. Therefore, more research could be carried out to explore into the etiology of MetS.

There are also some limitations to this study. First, analyses were performed using cross-sectional data. Therefore, a temporal relationship was established between the studied MetS components. Second, as the requirement of CFA, the entry could only be analyzed with no missing value. Third, as the lack of measurement of fasting insulin, HOMA-IR and other indicators were not available to build the mature models such as the Pladevall *et al.*’s, Li and Ford’s, and Martinez-Vizcaino *et al.*’s.

## Conclusions

Our study confirms that MetS is a multi-factorial syndrome, and it suggests that there could be some patterns of common causation for the core components of MetS. This study preformed CFA in a Chinese population in Beijing, and suggests that the single-factor model of metabolic syndrome with WC, TG, FPG and SBP is plausible in women of all age group, and fits men in young adulthood and senior as well. The model with WC, TG/HDL-C ratio, FPG and MAP has better fit in men of middle-age.

## Methods

### Survey methodology and laboratory tests

In 2005, a surveillance of risk factors for non-communicable diseases was conducted by Municipal Health Bureau and the Centers for Disease Control and Prevention (CDC) in Beijing, China. The cross-sectional study employed proportional multistage cluster random sampling design and selected 19,216 persons aged ≥ 18 year-old who have lived in the city for at least six months. The survey was carried out during August and September, 2005. It included questionnaires, anthropometric and blood pressure measures, blood biochemical analysis. The questionnaire was composed of demographic profile as age, gender, educational background; risk factors of non-communicable diseases including smoking habit, alcohol consumption, diet, and physical exercise; prevalence of non-communicable diseases such as hypertension, type 2 diabetes mellitus, dyslipidemia, and overweight or obesity. The anthropometric measurements included weight, height, waist and hip circumference; blood pressure include systolic and diastolic pressures; and the laboratory examinations included fasting plasma glucose, total cholesterol, high-density lipoprotein cholesterol (HDL-C), low-density lipoprotein cholesterol (LDL-C), and creatinine. Detailed survey methodology, measurements and laboratory tests and was depicted elsewhere in 2010 [31]. This study was approved by the ethics committee of the Capital Medical University of China, and performed in accordance with the principles of Declaration of Helsinki (reference number: 2013SY26).

### Inclusion criteria and layered approach

Primarily, there were 16,711 persons included in the database. 688 subjects with any missing value in sex, waist curriculum, systolic blood pressure, diastolic blood pressure, fasting plasma glucose, triglycerides, or HDL-C were excluded. 8,551 people with medication treatment within two weeks of tests were also excluded. At last, 7,472 individuals were remained under CFA. The age groups were divided as “18-34 years”,“35-59 years” and “over 60 years”, which stood for young, middle-aged, and senior population, and models were analyzed in each age-group. There were 1,038, 1,368 and 260 male subjects in young, middle-aged and senior age-group, respectively; and 1,573, 2,927 and 306 female subjects in each age-group, respectively.

### Statistical analysis

Continuous variables were expressed as means and standard deviations (SD), and discrete variables were presented as proportions. The study population met all requirements for factor analysis. In testing the normality assumption, five variables were found to have a high skewness – TG, TG/HDL-C ratio, FPG, SBP and MAP; these variables were transformed with a natural log function. The dichotomous variables used to define MetS risk factors were categorized by cut points of the latest definition [16]. The detailed diagnosis criteria are as follows:

▪ Obesity: ≥ 85 cm in men; ≥ 80 cm in women;

▪ Raised TG level (drug treatment for raised TG level is an alternative indicator): ≥ 150 mg/dL (1.7 mmol/L);

▪ Reduced HDL-C level (drug treatment for reduced HDL-C level is an alternative indicator): < 40 mg/dL (1.0 mmol/L) in men; < 50 mg/dL (1.3 mmol/L) in women;

▪ Raised blood pressure (antihypertensive drug treatment in a patient with a history of hypertension is an alternate indicator): SBP ≥ 130 and/or DBP ≥ 85 mmHg;

▪ Raised FPG level (drug treatment of raised glucose is an alternative indicator): ≥ 100 mg/dL (5.6 mmol/L);

▪ Participants fulfilling at least three out of these five components were diagnosed as having MetS.

The database was established by EpiData v3.02. The statistical analyses were performed using Statistical Package of Social Science for Windows v13.0 (SPSS Inc., Chicago, IL, USA). Statistical significance was set as *P* < 0.05.

To examine the construct validity of the two competing models for MetS, the CFA performed with maximum likelihood estimation using AMOS v7.0 (AMOS Development Co., Crawfordville, FL, USA).

### Consent

Written informed consent was obtained from the patient for publication of this report and any accompanying images.

## Abbreviations

MetS: Metabolic syndrome; WHO: World Health Organization; IDF: International diabetes federation; NCEP: National Cholesterol Education Program; ATP III: Adult Treatment Panel III; CDS: Chinese Diabetes Society; JCDCG: Joint Committee for Developing Chinese Guidelines on Prevention and Treatment of Dyslipidemia in Adults; WC: Waist circumference; TG: Triglycerides; SBP: Systolic blood pressure; DBP: Diastolic blood pressure; HDL-C: High density lipoprotein cholesterol; FPG: Fasting plasma glucose; EFA: Exploratory factor analysis; CFA: Confirmatory factor analysis; MAP: Mean artery pressure; HOMA-IR: Homeostasis model of assessment for insulin resistance; SRMR: Standardized root mean square residual; CFI: Comparative fit index; RMSEA: Root mean square error of approximation; GFI: Goodness of fit index; AIC: Akaike information criteria; BIC: Bayes information criterion; CAIC: Consistent version of AIC; ECVI: Expected cross-validation index; CDC: Centers for disease control and prevention; LDL-C: Low density lipoprotein cholesterol; SD: Standard deviation.

## Competing interests

The author declare that they have no competing interests.

## Authors’ contributions

XG and PZ designed the study; WW and DH participated in all data interpretation; DH drafted the manuscript; XL and QG carried out statistical analysis; LW, YL and YW critically reviewed the manuscript. All authors have read and approved the final manuscript.
